# Cost-effectiveness of group medical visits and microfinance interventions versus usual care to manage hypertension in Kenya: a secondary modelling analysis of data from the Bridging Income Generation with Group Integrated Care (BIGPIC) trial

**DOI:** 10.1016/S2214-109X(24)00188-8

**Published:** 2024-08

**Authors:** Junxing Chay, Rebecca J Su, Jemima H Kamano, Benjamin Andama, Gerald S Bloomfield, Allison K Delong, Carol R Horowitz, Diana Menya, Richard Mugo, Vitalis Orango, Sonak D Pastakia, Cleophas Wanyonyi, Rajesh Vedanthan, Eric A Finkelstein

**Affiliations:** Health Services and Systems Research, Duke–NUS Medical School, Singapore; Health Services and Systems Research, Duke–NUS Medical School, Singapore; School of Medicine, Moi University College of Health Sciences, Eldoret, Kenya; Academic Model Providing Access to Healthcare, Eldoret, Kenya; Duke Global Health Institute, Duke University, Durham, NC, USA; Center for Statistical Sciences, Brown University, Providence, RI, USA; Institute for Health Equity Research, Icahn School of Medicine at Mount Sinai, New York, NY, USA; School of Medicine, Moi University College of Health Sciences, Eldoret, Kenya; Academic Model Providing Access to Healthcare, Eldoret, Kenya; Academic Model Providing Access to Healthcare, Eldoret, Kenya; Department of Pharmacy Practice, Purdue University College of Pharmacy, West Lafayette, IN, USA; Academic Model Providing Access to Healthcare, Eldoret, Kenya; Department of Population Health, Grossman School of Medicine, New York University, New York, NY, USA; Health Services and Systems Research, Duke–NUS Medical School, Singapore; Duke Global Health Institute, Duke University, Durham, NC, USA

## Abstract

**Background:**

The Bridging Income Generation with Group Integrated Care (BIGPIC) trial in rural Kenya showed that integrating usual care with group medical visits or microfinance interventions reduced systolic blood pressure and cardiovascular risk in participants. We aimed to estimate the incremental cost-effectiveness of three BIGPIC interventions for a modelled cohort and by sex, as well as the cost of implementing these interventions.

**Methods:**

For this analysis, we used data collected during the BIGPIC trial, a four-group, cluster-randomised trial conducted in the western Kenyan catchment area of the Academic Model Providing Access to Healthcare. BIGPIC enrolled participants from 24 rural health facilities in rural western Kenya aged 35 years or older with either increased blood pressure or diabetes. Participants were assigned to receive either usual care, group medical visits, microfinance, or a combination of group medical visits and microfinance (GMV–MF). Our model estimated the incremental cost-effectiveness of the three BIGPIC interventions via seven health states (ie, a hypertensive state, five chronic cardiovascular-disease states, and a death state) by simulating transitions between health states for a hypothetical cohort of individuals with hypertension on the basis of QRISK3 scores. In every cycle, participants accrued costs and disability-adjusted life-years (DALYs) associated with their health state. Incremental cost-effectiveness ratios (ICERs) were calculated for the entire modelled cohort and by sex by dividing the incremental cost by the incremental effectiveness of the next most expensive intervention. The main outcome of this analysis was ICERs for each intervention evaluated. This analysis is registered at ClinicalTrials.gov (NCT02501746).

**Findings:**

Between Feb 6, 2017, and Dec 29, 2019, 2890 people were recruited to the BIGPIC trial. 2020 (69·9%) of 2890 participants were female and 870 (30·1%) were male. At baseline, mean QRISK3 score was 11·5 (95% CI 11·1–11·9) for the trial population, 11·9 (11·5–12·2) for male participants, and 11·3 (11·0–11·6) for female participants. For the population of Kenya, group medical visits were estimated to cost US$7 more per individual than usual care and result in 0·005 more DALYs averted (ICER $1455 per DALY averted). Microfinance was estimated to cost $19 more than group medical visits but was only estimated to avert 0·001 more DALYs. Relative to group medical visits, GMV–MF was estimated to cost $29 more and avert 0·009 more DALYs ($3235 per DALY averted). Relative to usual care, GMV–MF was estimated to cost $37 more and avert 0·014 more DALYs ($2601 per DALY averted). In the first year of the intervention, usual care was estimated to be the least expensive intervention to implement ($87 per participant; $10 238 per health-facility catchment area [HFCA]), then group medical visits ($99 per participant; $12 268 per HFCA), then microfinance ($120 per participant; $14 172 per HFCA), with GMV–MF estimated to be the most expensive intervention to implement ($139 per participant; $16 913 per HFCA).

**Interpretation:**

Group medical visits and GMV–MF were estimated to be cost-effective strategies to improve blood-pressure control in rural Kenya. However, which intervention to pursue depends on resource availability. Policy makers should consider these factors, in addition to sex differences in programme effectiveness, when selecting optimal implementation strategies.

**Funding:**

US National Institutes of Health.

## Introduction

Cardiovascular disease constitutes approximately a third of deaths worldwide, with 80% of deaths from cardiovascular disease occurring in low-income and middle-income countries (LMICs).^1^ Hypertension is a leading risk factor for cardiovascular disease and is one of the most modifiable due to the widespread availability of low-cost and effective medications. However, the prevalence of hypertension continues to increase in LMICs, where blood-pressure control is low.^2^

In Kenya, almost a third of adults have hypertension.^3^ Despite the availability of low-cost antihypertensive treatments, many adults remain untreated and only half of those receiving treatment have adequate blood-pressure control.^4^ This low rate of treatment could be attributable to socioeconomic challenges such as inequitable access to health care, inability to afford medication, little education and awareness, or high opportunity costs of seeking treatment.^5,6^

In 2021, we reported the efficacy of a large, community-based, cluster-randomised trial in Kenya that was named Bridging Income Generation with Group Integrated Care (BIGPIC).^7^ In 2890 individuals, integrating usual care for hypertension with group medical visits, microfinance, or a combination of group medical visits and microfinance (GMV–MF) improved systolic blood pressure (SBP) and overall cardiovascular risk more so than usual care.^7^ Furthermore, in all groups, female participants had greater SBP reductions than male participants.^7^

Although these interventions showed potential in reducing SBP compared with usual care, their cost-effectiveness and affordability were not evaluated. We aimed to estimate the incremental cost-effectiveness of the three BIGPIC interventions for a modelled cohort and by sex, as well as the cost of implementing these interventions.

## Methods

### Study design and participants

For this analysis, we predominantly used data collected during the BIGPIC trial. The BIGPIC trial was a four-group, cluster-randomised trial conducted in the western Kenyan catchment area of the Academic Model Providing Access to Healthcare (AMPATH) chronic disease-management programme, a multicomponent, facility-based hypertension and diabetes management programme.^8^ AMPATH provided usual care, which met criteria for the task-shifted model of chronic-disease management recommended by the Kenyan Ministry of Health.^9^

BIGPIC enrolled participants from 24 rural health facilities in rural western Kenya between Feb 6, 2017, and Dec 29, 2019, all of whom were aged 35 years or older and had either increased blood pressure (ie, SBP ≥140 mm Hg or diastolic blood pressure [DBP] ≥90 mm Hg) or diabetes (ie, fasting glucose ≥7 mmol/L or random glucose ≥11·1 mmol/L).^10^ Health facilities were clustered into four trial groups (ie, usual care, microfinance, group medical visits, and GMV–MF). Further detail has been reported previously.^7,10^

Participants provided written informed consent for the trial and all subsequent analyses.

The BIGPIC protocol was approved by the institutional review boards of Moi University College of Health Sciences (Eldoret, Kenya), the Icahn School of Medicine at Mount Sinai (New York, NY, USA), and the Grossman School of Medicine (New York University, New York, NY, USA). Ethics approval was provided for both the trial and this analysis. This Article was written in accordance with the CHEERS guideline (appendix pp 3–5).^11^

This analysis is registered at ClinicalTrials.gov (NCT02501746).

### Procedures

Group medical visits incorporated group-based care and health education to improve chronic-disease management and preventive care.^12^ Participants assigned to group medical visits joined group meetings once per month, which consisted of individual consultations with a clinician and a group discussion led by community health workers about a self-care or a health-education topic. Participants assigned to the microfinance intervention received usual care and met once per month in community savings groups to manage their savings and contribute to a social fund for emergency or welfare purposes, with the intervention providing interest-bearing loans to group members. Participants assigned to GMV–MF joined group meetings once per month, which consisted of an initial microfinance intervention and then clinical care in the form of group medical visits once per month. Usual care consisted of AMPATH’s multicomponent chronic disease-management programme, which used medicines contained in the Kenyan national formulary and included both pharmacological interventions (eg, medications for hypertension and diabetes, blood tests, and urine analysis) and non-pharmacological interventions (eg, blood-pressure screening and education for management of hypertension and diabetes).

Interventions were conducted for 1 year and participants were not charged for clinic visits. Usual care and the cost of medication was the same for participants across groups.

### Data sources

We developed a Markov model in TreeAge Pro version R2 to simulate transitions between health states for a hypothetical cohort of individuals with hypertension on the basis of QRISK3 scores. Using annual cycles, we estimated costs and utility decrements associated with cardiovascular events during the next 10 years after the intervention. We based the characteristics of our cohort on the BIGPIC trial participants.^7^ Our model used a hypothetical cohort of individuals aged 61 years, the mean age of BIGPIC participants, who had been diagnosed with hypertension or diabetes but had no history of cardiovascular disease.

GMV–MF was the only effective intervention relative to usual care in the original BIGPIC trial; group medical visits and microfinance were not effective. As group medical visits and microfinance were not more effective than usual care, we also calculated the average cost-effectiveness ratio of GMV–MF relative to usual care.

QRISK3 scores were calculated individually for each participant on the basis of collected participant data in the BIGPIC trial. They measure an individual’s risk of having a heart attack or stroke in the next 10 years and are based on age, sex, ethnicity, and clinical information (eg, SBP, diabetes, and cholesterol).^13^ Blood pressure and hypertension data were collected by an AMPATH physician using standard medical protocols. Sex data were self-reported; the options were male or female. Cardiovascular events were defined as either a heart attack or a stroke, two of the most common major acute cardiovascular events associated with hypertension.^14^

Data quality was vetted internally by AKD, VO, and RM (appendix p 6).

### Model structure

**Figure 1:**
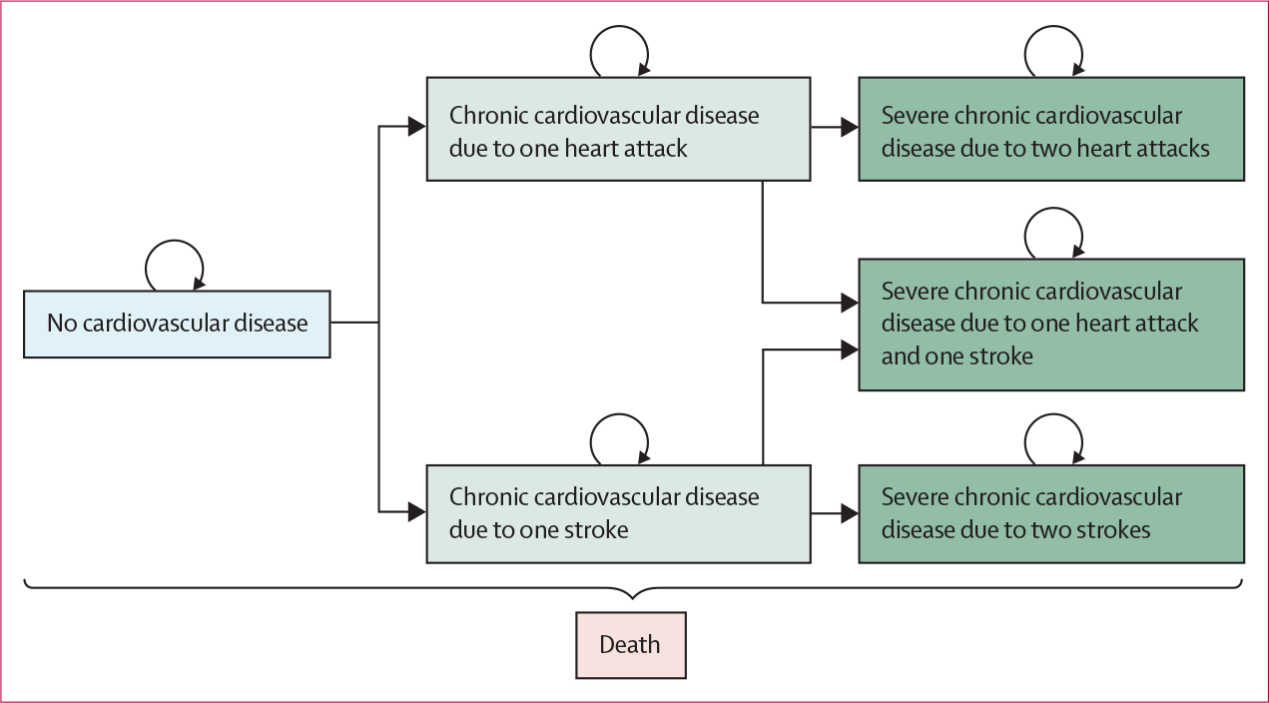
Markov diagram Rectangles show exclusive health states. Curved arrows indicate transitions to the same health state. Straight arrows indicate transitions to other health states. Transitions to the death state could occur from any state.

Our model to estimate the incremental cost-effectiveness of the three BIGPIC interventions consisted of seven health states (ie, a hypertensive state; five chronic cardiovascular-disease states, which were defined by the number and type of cardiovascular-disease events that a participant had had; and a death state; figure 1). All participants began in the hypertensive state after the end of their assigned intervention and incurred intervention costs. For simplicity, we assumed that all individuals did not have a heart attack or stroke during the intervention period. For every cycle that a participant remained in the hypertensive state, they incurred the cost of hypertension medication and the disability-adjusted life-years (DALYs) of having hypertension or diabetes.

In each annual cycle of the model, individuals had the risk of heart attack or stroke, which could have been fatal, and the risk of dying from non-cardiovascular-disease causes. Heart attacks and strokes incurred a hospitalisation (ie, treatment in hospital) cost of 14 days and a DALY decrement of 28 days. Individuals who lived after a heart attack or stroke transitioned to one of the chronic cardiovascular-disease states on the basis of their history, in which they incurred the cost of chronic cardiovascular-disease management and associated DALYs. As risk of cardiovascular-disease events increases with previous history of cardiovascular disease, individuals in chronic states had increased risk of a second or third cardiovascular-disease event in subsequent cycles. For tractability, our model allowed for a maximum of two non-fatal cardiovascular-disease events; a third event was assumed to be fatal. Individuals in the death state incurred zero costs and a DALY of 1 for each cycle until the end of the model. The parameter inputs and ranges of values used for analysis for our model are provided (table 1).

Our model structure was consistent with previously published cost-effectiveness studies evaluating hypertension interventions.^21–23^

## Transition probabilities

We derived the annual probability of a first cardiovascular-disease event from QRISK3 scores. QRISK3 scores were calculated at baseline and after the BIGPIC intervention completion. Effects of an intervention on QRISK3 scores were modelled via linear mixed-effects models, with random effects used to consider clustering of individuals in health-facility catchment areas. Control variables included the baseline covariates age, sex, recruitment pathway (ie, newly screened, previously screened but not linked to care, active in care for less than 6 months, previously in care but no clinic visit in past 6 months, and currently in private care but wishing to transfer to the public sector care system), amount of pre-trial microfinance activity in the cluster at baseline, type of health facility (ie, dispensary, health centre, or subcounty hospital), cluster-specific mean of outcome, and value of the outcome.^7^ QRISK3 scores for the usual-care group were based on mean QRISK3 scores at baseline. QRISK3 scores for groups receiving microfinance, group medical visits, or GMV–MF were calculated by subtracting difference-in-differences estimates from the baseline score. Similar to previous studies,^24,25^ 10-year risks were annualised, assuming a constant hazard for 10 years (appendix p 7).

On the basis of local data, we assumed that 60% of cardiovascular-disease events were strokes and 40% were heart attacks.^14^ The probability of surviving a stroke or a heart attack were based on people who had presented at Kenyatta National Hospital (Nairobi, Kenya) and Moi Teaching and Referral Hospital (Eldoret, Kenya).^19,20^

As QRISK3 scores were only validated for people with no previous cardiovascular-disease history, probabilities for subsequent cardiovascular-disease events were estimated by adjusting probabilities of first events with hazard ratios, which compared the risks of participants with cardiovascular-disease history with those without.^18^ We multiplied the implied hazard rates of the first cardiovascular-disease event and related mortality rate by the hazard ratios of participants with a history of stroke only, heart attack only, or both heart attack and stroke to derive the annual probability of a subsequent heart attack or stroke. We used the same method to obtain survival probabilities for subsequent cardiovascular-disease events (appendix pp 8–10). Kenyan life tables were used to inform age-specific probabilities of dying from non-cardiovascular-disease causes.^17^

### Costs and disability weights

Costs were considered from the perspective of the health-care system in Kenya and included the costs of interventions, medical costs related to hypertensive and chronic-disease states, and hospitalisation costs. We used the activity-based costing approach^26^ to track intervention costs prospectively using standard cost-collection instruments, such as validated cost tracking forms and questionnaires, which captured all relevant labour, materials, supplies, and contracted-services costs to deliver the interventions, including administration and oversight, clinician and field-staff training, participant training, baseline screening, confirmatory tests, implementation, quarterly reviews, and usual-care activities. The costs of each intervention per person were obtained by summing costs of activities associated with each intervention group and dividing by the number of participants in that group. Costs related to hypertension and chronic cardiovascular-disease management were estimated by clinicians who were familiar with the local health-care system, including RV, and included medications, clinic visits, laboratory tests, and electrocardiograms. Hospitalisation costs for people who had had heart attacks or strokes were obtained from Subramanian and colleagues.^15^ Costs were captured in 2020 Kenyan shillings and converted to US$ with the 2020 exchange rate of US$0·0093 per Kenyan shilling (table 1; appendix p 11).

Disability weights were obtained from the Global Burden of Disease Study 2019.^16^ We identified appropriate disability weights using provided health-state names and simple descriptions in consultation with RV, a cardiologist from the BIGPIC trial. If a disability weight was not available for a health state, we used disability weights for similar health conditions (appendix p 12), in consultation with clinicians, including RV. The disability weight for the state of having no cardiovascular disease (ie, the hypertensive state) was calculated as the prevalence-weighted mean of diabetes and hypertension disability weights, representing the characteristics of participants in the BIGPIC trial. Disability weights for heart attacks were based on acute myocardial infarction and disability weights for strokes were based on acute ischaemic stroke. Disability weights for the states of chronic cardiovascular disease due to heart attack were based on disability weights for angina and heart failure due to ischaemic heart disease. Disability weights for the states of chronic cardiovascular disease due to stroke were based on disability weights for chronic ischaemic stroke (appendix p 12).

### Cost-effectiveness analysis

Incremental cost-effectiveness ratios (ICERs) were calculated for the entire modelled cohort and by sex by dividing the incremental cost by the incremental effectiveness of the next most expensive intervention. This ratio presented the additional cost required to avert one DALY relative to the next most expensive intervention. As there is currently no consensus on what the cost-effectiveness threshold for health interventions in Kenya should be, we considered which interventions were cost-effective at various willingness-to-pay (WTP) thresholds.

We discounted costs and benefits at 3% per annum beyond the first year, and applied half-cycle correction to account for uncertainty in the timing of transitions within a cycle.

### Statistical analysis

The main outcome of this analysis was ICERs for each intervention evaluated. Uncertainty and heterogeneity of the data were accounted for via deterministic and probabilistic sensitivity analyses. We investigated the sensitivity of base case results for the entire modelled cohort by extending the time to 20 years—the expected lifetime of the cohort.^27^ In the absence of information of risk for the next 10 years, we assumed that the probability of having a cardiovascular-disease event did not change from base case during 20 years. Furthermore, we investigated the cost-effectiveness of a scaled-up version of all three BIGPIC interventions, in which interventions were ongoing for 10 years for the population.

We conducted sensitivity analyses using results for the entire modelled cohort only. We conducted a one-way deterministic sensitivity analysis to identify the most influential parameters on incremental net monetary benefits (INMBs) on the basis of a WTP threshold of two times the gross domestic product (GDP) per capita in Kenya, as plausible values for intervention effectiveness were small and included zero and negative values (table 1). We also conducted probabilistic sensitivity analysis with 10 000 iterations to evaluate the effects of statistical uncertainty of parameter values on ICERs. QRISK3 score differences were taken from normal distributions, cost parameters were taken from γ distributions, and disability weights were taken from β distributions. Values for hazard ratios, the proportion of cardiovascular-disease events that were strokes, and the proportion of fatal cardiovascular-disease events were taken from log-normal distributions. Distributions were chosen to reflect the nature of the parameter and feasible values while adhering to best practice.^28^

To parameterise distributions, we used the base case value as the mean and corresponding published SE for SD, if available (table 1). SEs for intervention effectiveness and hazard ratios were derived from published 95% CIs (table 1). If SEs were unavailable, we used 10% of the base case value. Results of the probabilistic sensitivity analysis were presented as cost-effectiveness acceptability curves that showed each intervention’s probability of being optimal (ie, the highest net monetary benefit) at different WTP thresholds.

For individuals who were lost to follow-up, their QRISK scores were regarded as missing and were removed from the analysis.

Missing data were removed from analysis.

Data collected for the trial were processed and analysed with R version 4.0.0. The modelling analysis was conducted using TreeAge Pro version R2.

### Role of the funding source

The funder of the the original BIGPIC trial and this analysis had no role in study design, data collection, data analysis, data interpretation, or writing of the report.

## Results

Between Feb 6, 2017, and Dec 29, 2019, 2890 people were recruited to the BIGPIC trial. 2020 (69·9%) of 2890 participants were female and 870 (30·1%) were male. At baseline, mean QRISK3 score was 11·5 (95% CI 11·1–11·9) for the trial population, 11·9 (11·5–12·2) for male participants, and 11·3 (11·0–11·6) for female participants.

For the entire modelled cohort, usual care was estimated to be the least expensive intervention, then group medical visits, then microfinance, with GMV–MF estimated to be the most expensive. Group medical visits were estimated to cost $7 more per individual than usual care and result in 0·005 more DALYs averted (ICER $1455 per DALY averted; table 2). Microfinance was estimated to cost $19 more than group medical visits but was only estimated to avert 0·001 more DALYs. As a result, microfinance was extended dominated, meaning that a combination of other alternative interventions (ie, group medical visits and GMV–MF) could lead to greater DALY reductions at equal or lower cost. Relative to group medical visits, GMV–MF was estimated to cost $29 more and avert 0·009 more DALYs ($3235 per DALY averted). Relative to usual care, GMV–MF was estimated to cost $37 more and avert 0·014 more DALYs ($2601 per DALY averted).

For female participants, microfinance was dominated, meaning that a single alternative intervention could produce the same or greater DALY reductions at equal or lower cost. For male participants, microfinance was extended dominated. For female participants, group medical visits were estimated to have an ICER of $311 per DALY averted relative to usual care, whereas GMV–MF was estimated to have an ICER of $5480 per DALY averted relative to group medical visits and an ICER of $2364 per DALY averted relative to usual care. For men, GMV–MF was estimated to have an ICER of $3762 per DALY averted relative to usual care, which was the next most expensive non-dominated intervention, as group medical visits were dominated by usual care (appendix p 13).

In the first year of the intervention, usual care was estimated to be the least expensive intervention to implement ($87 per participant; $10 238 per health-facility catchment area [HFCA]), then group medical visits ($99 per participant; $12 268 per HFCA), then microfinance ($120 per participant; $14 172 per HFCA), with GMV–MF estimated to be the most expensive intervention to implement ($139 per participant; $16 913 per HFCA). Assuming the same enrolment in each subsequent year, usual care was estimated to be the least expensive intervention to implement ($67 per participant; $7862 per HFCA), then microfinance ($67 per participant; $7946 per HFCA), then group medical visits ($71 per participant; $8749 per HFCA), with GMV–MF estimated to be the most expensive intervention to implement ($72 per participant; $8832 per HFCA; appendix p 11).

A 20-year time for the model increased estimated total costs and DALYs for all interventions (appendix p 14). However, relative to base case, incremental costs between non-dominated interventions were estimated to be lower and incremental DALYs averted were estimated to be higher due to more cardiovascular events estimated to be avoided. As a result, group medical visits (ICER $372 per DALY averted) and GMV–MF ($1078 per DALY averted) were estimated to have lower ICERs than base case. Microfinance remained extended dominated.

When including recurrent costs of the intervention for 10 years, which could be required to maintain reduced cardiovascular-disease risk, micro finance was estimated to become less expensive than group medical visits due to smaller recurring costs (ICER $4868 per DALY averted) and, as a result, dominated group medical visits because it averted 0·001 more DALYs. The ICER for GMV–MF ($6634 per DALY averted) relative to microfinance instead of group medical visits, was estimated to be larger than base case as recurrent costs amplified cost differences.

**Figure 2:**
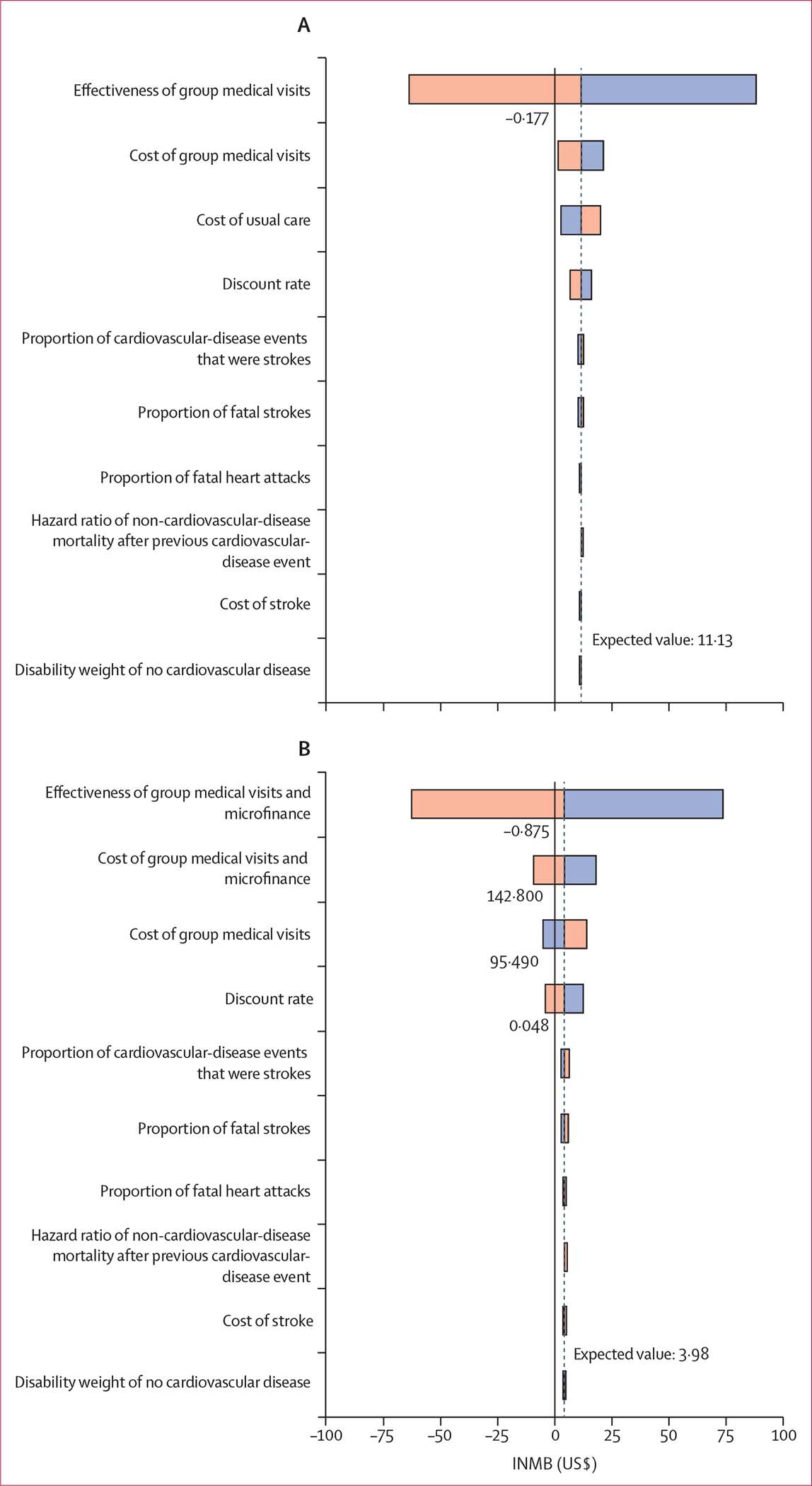
Tornado diagram of the ten most influential model parameters in the base case analysis (A) Effects of parameter variation on INMBs, in US$, for group medical visits relative to usual care. (B) Effects of parameter variation on INMBs, in US$, for GMV–MF relative to usual care. Bars indicate the range of INMB values corresponding to sensitivity ranges. Grey line indicates base case INMB value. Orange bars indicate when parameters are increasing from their base case values. Blue bars indicate when parameters are decreasing from their base case values. GMV–MF=group medical visits and microfinance. INMB=incremental net monetary benefit.

The influence of model parameters on INMBs for non-dominated interventions for the entire modelled cohort by sex were examined in the deterministic one-way sensitivity analyses (figure 2). Reduction in QRISK3 scores was estimated to be most influential on INMBs. Group medical visits and GMV–MF were estimated to no longer be incrementally cost-effective if QRISK3 scores for group medical visits were reduced by 46% (−0·177) and if QRISK3 scores for GMV–MF were reduced by 6% (−0·875). Variations in the remaining model parameters did not change estimates of base case results for group medical visits. However, the cost of GMV–MF relative to group medical visits and the discount rate were estimated to be influential on the cost-effectiveness of GMV–MF relative to group medical visits (appendix pp 15–16).

**Figure 3:**
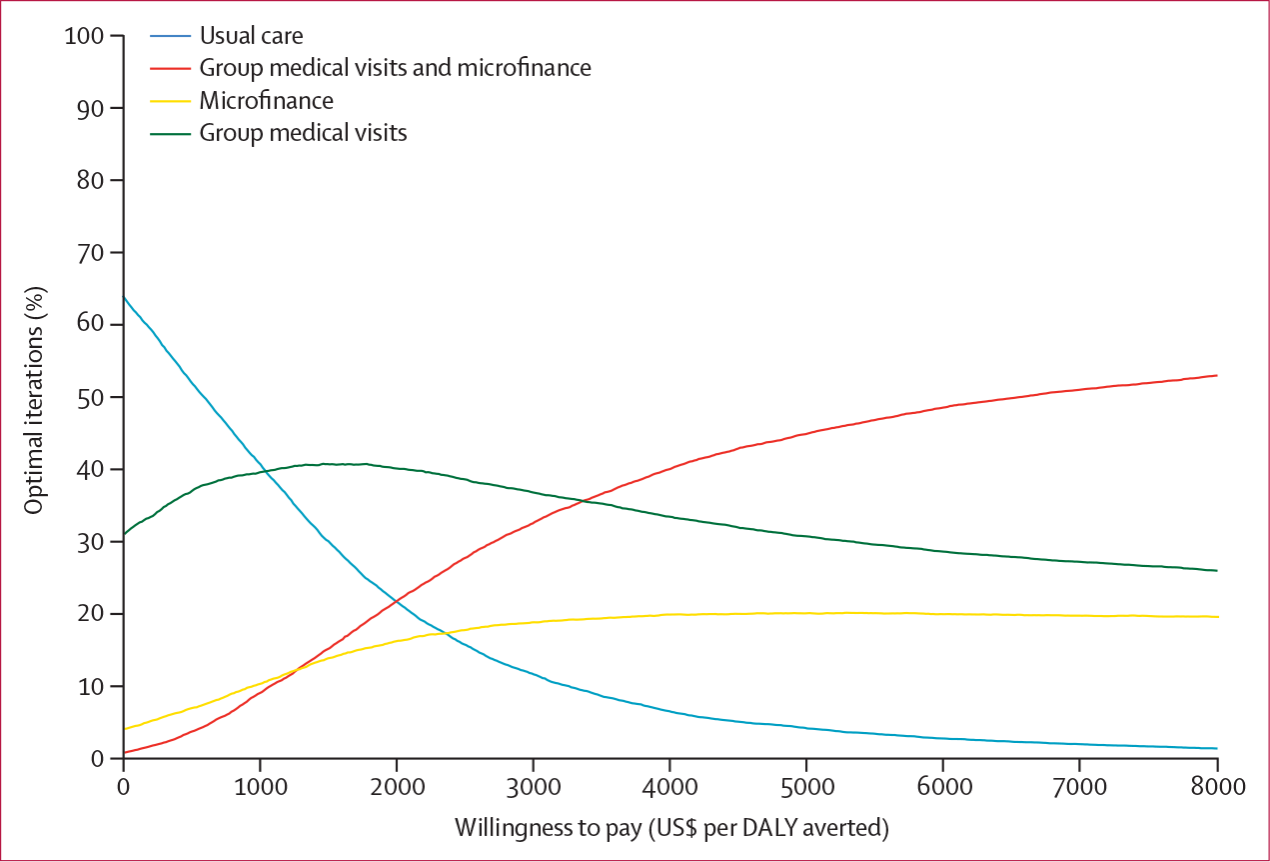
Cost-effectiveness acceptability curves for the population base case analysis DALY=disability-adjusted life-year.

Results from the probabilistic sensitivity analysis were estimated to be in line with base case (figure 3). At a WTP threshold less than $1040 per DALY averted (ie, less than one times GDP per person), usual care was estimated to be the intervention most likely to be optimal. However, offering group medical visits to only female participants was cost-effective below this threshold (table 2). When WTP was between $1040 and $3360 per DALY averted (ie, within the ranges of one times and two times GDP per person), group medical visits were estimated to be the intervention most likely to be optimal. GMV–MF was estimated to be the intervention most likely to be optimal above a WTP threshold of $3360 per DALY averted.

## Discussion

We estimated that integrating group medical visits and GMV–MF into usual care could be cost-effective strategies to control blood pressure in rural Kenya. For the entire modelled cohort, group medical visits were estimated to be the optimal strategy when WTP was between $1040 and $3360 per DALY averted. GMV–MF was estimated to be the optimal strategy above a WTP threshold of $3360 per DALY averted. However, at the lowest WTP threshold, usual care was estimated to be the intervention most likely to be optimal. Our results were most sensitive to the extent to which an intervention reduced cardiovascular-disease risk.

Stratified analyses showed that the estimated cost-effectiveness of each intervention differed by sex, as male and female participants had differential benefit (eg, we estimated that male participants benefited from microfinance but did not benefit from group medical visits). These findings suggest that a non-stratified approach to hypertension management could be suboptimal. Although the BIGPIC trial was not powered to detect a difference in intervention effectiveness between male participants and female participants, our results suggest a sex-based implementation strategy could be optimal, which should be an area of future research. An example of a successful gender-based approach is the US Center for Disease Control and Prevention’s WISEWOMAN programme, which expanded the services of existing US federal programmes to improve cardio vascular health in women who were uninsured and had low income and cardiovascular-disease risk factors in 24 US states.^29^ Through this programme, women at risk of cardiovascular disease could access screening, lifestyle programmes, medication, and referral services. In a 2006 analysis assessing the cost-effectiveness of WISEWOMAN, participants had significantly improved SBP, DBP, and 10-year risk of coronary heart disease.^30^ Moreover, differentiated service-delivery programmes have been implemented for HIV care in many countries, including Kenya, and several gender-based models have been implemented.^31–33^ The benefits of these service-delivery programmes have included improved HIV-cascade outcomes (eg, numbers of people screened, on treatment, and with suppressed viral load) and reduced or equivalent cost, among others. Hypertension and HIV care share similarities in that both require primary-care services and lifelong support; a similar approach could be considered for hypertension care.^31^

Our analysis was in accordance with the CHEERS guideline for reporting uncertainty in economic evaluations,^11^ which recommends conducting economic evaluations even when the primary outcome of interest might not be statistically different between groups on the basis of a conventional p value cutoff. As a result, an intervention can be both cost-effective on the basis of established thresholds of cost per quality-adjusted life-year gained and not statistically different in terms of the primary outcome of interest. This approach is consistent with 2019 recommendations to stop using strict p value cutoffs and focus on the totality of evidence, recognising that failure to reject the null hypothesis is not synonymous with an intervention being ineffective.^34^ In our analysis, we addressed uncertainty with one-way sensitivity analyses and cost-effectiveness acceptability curves, which incorporated uncertainty in the primary outcome and all parameters of interest. Policy makers should consider both the base case results and sensitivity analyses when interpreting the value of implementing the three BIGPIC interventions.

BIGPIC focused on promoting access to care by improving economic stability, reliance on communities as social support, and rural populations. As components of BIGPIC were integrated into existing public-sector health-care infrastructure and relied on local community health workers, the effectiveness and cost-effectiveness of BIGPIC interventions could be generalisable to rural settings in other LMICs that have similar populations and public-sector health settings, such as other countries in Africa. Our approach to group medical visits, particularly group-based educational discussions of chronic-disease management and the provision of individual clinical sessions with medical staff, are similar to other settings, such as the USA.^35,36^ However, as our results were based on data from Kenya only, particularly in terms of health-care costs, local adaptations will be required to establish the economic value of implementing the three BIGPIC interventions in other countries and settings.

Our findings focused solely on the estimated costs of health benefits of the interventions, but group medical visits and microfinance might also confer non-health benefits. For example, microfinance can result in improved economic wellbeing, increased empowerment of female participants, and reduced HIV risk behaviours; group medical visits can improve trust, social support, and social cohesion among community members.^37–39^ Furthermore, microfinance initiatives could improve access to financial credit for vulnerable populations, such as people with children,^40,41^ potentially reducing financial barriers and improving health-care access.^42^ Groups that aimed to increase appropriate care-seeking, home-prevention, and care practices for mothers and newborns in Bangladesh, India, Malawi, and Nepal were shown to cost-effectively improve maternal and neonatal mortality and reduce stillbirths.^43^ In rural Kenya, a group-based parenting intervention to promote early childhood development was also shown to be cost-effective.^44^ The integration of both strategies in the form of GMV–MF could complement each other and result in further improvements in quality of life. Future analyses will need to be conducted to understand the total health, economic, and social benefits of these strategies.

Our findings were largely consistent with previous evaluations of hypertension-management interventions in LMICs. In rural Bangladesh, Pakistan, and Sri Lanka, home visits and lifestyle monitoring by trained community health workers, physician training, and public-sector care coordination reduced SBP by 5·2 mm Hg and were cost-effective in all three countries at three times GDP per capita thresholds or less.^45,46^ In Nepal, a community-based, multicomponent intervention consisting of blood-pressure monitoring and lifestyle counselling led by community health workers resulted in a reduced mean SBP of 4·9 mm Hg among participants with hypertension, and was cost-effective at a threshold of one times the GDP per capita.^47,48^ Although differences in implementation and context make direct comparison difficult, our analysis suggests that the three BIGPIC interventions have similar ICERs to these interventions and are cost-effective according to the WTP thesholds of these different countries.^49^ However, many countries, especially LMICs, apply lower thresholds.^50,51^ Whether or not BIGPIC is cost-effective depends both on the ICER and the threshold value, a consideration when assessing whether to implement the three BIGPIC interventions in other locations.

Our analysis has several strengths. First, intervention costs were tracked prospectively with the activity-based costing method, allowing for more accurate cost estimates. Second, we modelled stroke and heart-attack events, including costs and DALYs, separately when estimating the effect of the interventions on cardiovascular-disease events. Third, our model allowed for repeated cardiovascular-disease events and modelled worsening chronic conditions with each subsequent event, capturing the effects of the interventions on preventing multiple cardiovascular-disease events. Finally, our Markov-modelling approach is an improvement on previous studies^46,48^ that relied solely on static relationships between blood-pressure reductions and DALY improvements.

There were several limitations in our design, modelling assumptions, and analysis. First, although we report results by sex and suggest a sex-based implementation approach, the BIGPIC trial was not powered to detect a difference in intervention effectiveness between male and female participants. Second, our model assumed a constant hazard rate of having a cardiovascular-disease event during 10 years. If hazard increased over time, more cardiovascular-disease events would occur later and be affected by greater discounting than was estimated in the base case analysis. Cost and DALYs associated with cardiovascular disease would also be lower than was estimated in the base case analysis, as individuals would spend less time in cardiovascular-disease states. These variations would not affect incremental results if the hazard continued in the same away across comparators. However, if hazards increased more in the intervention groups relative to usual care (eg, the benefits of an intervention were front-loaded and not equally distributed across time), ICERs would be lower than base case ICERs (ie, more cost-effective). Third, assuming that the probability of having a cardiovascular-disease event did not change from base case during 20 years probably underestimated lifetime cardiovascular-disease risk, as risk is likely to increase over time due to age and worsening comorbidities, but the effect on incremental cost-effectiveness is likely to be negligible as underestimation applies to all comparators and is discounted more heavily than outcomes in analyses with shorter timeframes, such as the 10-year analysis. Fourth, due to scarce cost information, we assumed that the cost of hospitalisations for cardiovascular-disease events and of chronic cardiovascular-disease management were the same regardless of the number of cardiovascular-disease events a participant had. If people who had repeat cardiovascular-disease events have higher subsequent costs than we estimated in the base case, our ICERs would be conservative. Fifth, our analysis was limited to 10 years. Although modelling for lifetime cardiovascular-disease risk would have been ideal to capture the full benefits of the three BIGPIC interventions, cardiovascular-disease risk predictions beyond 10 years are challenging due to changes in risk factors and evolving treatments. When projecting outcomes during 20 years, we assumed that annual cardiovascular-disease risk was the same as the first 10 years, which probably underestimated lifetime risk. If intervention effectiveness is proportional to cardiovascular-disease risk, increased cardiovascular-disease risk results in increased absolute risk reductions and thus increased incremental effectiveness of the intervention. Finally, we only considered the effect of reduced hypertension on cardiovascular-disease risk. This assumption is also conservative, as improved hypertension management also reduces the risk of other chronic diseases that were not considered in this analysis.

Although the majority of rural areas in Kenya still have considerable gaps in terms of health-care resources and insurance,^52^ AMPATH was in accordance with the task-shifted model of chronic-disease management recommended by the Kenyan Ministry of Health^53^ and this work done in partnership with the Kenyan Ministry of Health.^54^ Furthermore, with Kenya’s 2023 transition from the National Hospital Insurance Fund to the Social Health Insurance Fund,^55^ there could be an opportunity to implement community-centred models, such as BIGPIC, in the near future. As chronic-disease management and primary-care approaches in public-sector health systems are increasingly being implemented in several countries,^56,57^ the implications of our analysis are relevant to both current and anticipated future health-system characteristics.

To our knowledge, ours is the first economic evaluation of integrating group medical visits and microfinance strategies into standard care for individuals with hypertension in Kenya. Our results suggest that, at common thresholds for cost-effectiveness, group medical visits and microfinance interventions could be cost-effective strategies to improve blood-pressure control and reduce morbidity and mortality related to cardiovascular disease in rural communities in Kenya. However, policy makers should consider sex differences in effectiveness when selecting optimal implementation strategies.

## Supplementary Material

1

## Figures and Tables

**Table 1:** Model inputs

	Base values	Deterministic sensitivity range

**QRISK3 score at baseline** ^7^		
Population	11·5	..
Men	11·9	..
Women	11·3	..
**QRISK3 score change relative to baseline for the population^7^**		
Microfinance	−0·41	−1·4 to 0·5
Group medical visits	−0·33	−4·4 to 0·7
Group medical visits and microfinance	−0·93	−4·9 to 0·0
**QRISK3 score change relative to baseline in men** ^7^		
Microfinance	−0·12	..*
Group medical visits	0·40	..*
Group medical visits and microfinance	−0·70	..*
**QRISK3 score change relative to baseline in women** ^7^		
Microfinance	−0·52	..*
Group medical visits	−0·60	..*
Group medical visits and microfinance	−1·00	..*
**Annual cost** ^7^		
Usual care	$87	±10%
Microfinance	$120	±10%
Group medical visits	$99	±10%
Group medical visits and microfinance	$139	±10%
Hypertension management	$68	±10%
Chronic cardiovascular-disease management	$125	±10%
Heart attack^15^	$1996	±10%
Stroke^15^	$1874	±10%
**Health-state disability weights** ^16^		
No cardiovascular disease	0·06	±10%
Chronic cardiovascular disease from one heart attack	0·08	±10%
Chronic cardiovascular disease from one stroke	0·14	±10%
Chronic cardiovascular disease from two heart attacks	0·17	±10%
Chronic cardiovascular disease from two strokes	0·49	±10%
Chronic cardiovascular disease from one heart attack and one stroke	0·33	±10%
**Disutility of cardiac events** ^16 †^		
Heart attack	0·01	±10%
Stroke	0·01	±10%
**Annual age-specific all-cause mortality rates** ^17^		
60–64 years	0·02	..
65–69 years	0·03	..
70–74 years	0·0	..
75–79 years	0·07	..
80–84 years	0·11	..
>85 years	0·20	..
**Hazard ratios** ^18^		
Heart attack after previous heart attack	1·42	1·20 to 1·69
Stroke after previous stroke	2·89	2·37 to 3·53
Heart attack after previous stroke	1·00	1·00 to 1·32
Stroke after previous heart attack	1·00	1·00 to 1·23
Heart attack after previous heart attack and stroke	1·95	1·37 to 2·8
Stroke after previous heart attack and stroke	3·13	2·22 to 4·43
Fatal heart attack after previous heart attack	1·22	1·0 to 1·43
Fatal heart attack after previous stroke	1·00	1·03 to 1·43
Fatal stroke after previous heart attack	1·22	1·00 to 1·35
Fatal stroke after previous stroke	1·00	1·00 to 1·35
All-cause death after previous cardiac event (assumption)	1·00	1·00 to 1·50
**Other parameters**		
Proportion of cardiovascular-disease incidence that is strokes (assumption)	0·60	0·5 to 0·7
Proportion of fatal heart attacks^19^	0·45	±10%
Proportion of fatal strokes^20^	0·45	±10%
Discount rate (assumption)	0·03	0·00 to 0·07

Costs are reported per person in 2020 US$.

* Data are available in the appendix (p 12).

† Disability weights of cardiac events were weighted to consider the duration of symptoms.

**Table 2:** Costs, DALYs, and incremental cost-effectiveness ratios of interventions in the Bridging Income Generation with Group Integrated Care trial during the next 10 years

	Total cost	Incremental cost	Total DALYs	Incremental DALYs averted	Incremental cost-effectiveness ratio

**Population**					
Usual care	$793	..	1·560	..	..
Group medical visits	$800	$7	1·555	0·005	$1455
Microfinance	$819	$19	1·554	0·001	Extended dominated*
Group medical visits and microfinance	$830	$29	1·546	0·009	$3235
**Men**					
Usual care	$800	..	1·566	..	..
Group medical visits	$819	$20	1·572	−0·006	Dominatedt
Microfinance	$831	$31	1·564	0·002	Extended dominated*
Group medical visits and microfinance	$840	$40	1·555	0·011	$3762
**Women**					
Usual care	$790	..	1·557	..	..
Group medical visits	$793	$3	1·548	0·009	$311
Microfinance	$814	$21	1·549	−0·001	Dominatedt
Group medical visits and microfinance	$825	$33	1·542	0·006	$5480

Costs are reported per person in 2020 US$. DALY=disability-adjusted life-year.

* A combination of other evaluated interventions could lead to greater DALY reductions at equal or lower cost.

† A single alternative intervention could produce the same or greater DALY reductions at equal or lower cost.

## Data Availability

De-identified participant data used in this analysis are available at https://pubmed.ncbi.nlm.nih.gov/33888251/. The Bridging Income Generation with Group Integrated Care trial protocol is available at https://pubmed.ncbi.nlm.nih.gov/28577673/. The TreeAge model will not be shared.

## Research in context

### Evidence before this study

Hypertension is a major risk factor for cardiovascular diseases, the leading cause of premature mortality globally. The Bridging Income Generation with Group Integrated Care (BIGPIC) trial showed that community-based interventions targeting financial and social barriers to hypertension care could reduce hypertension disease burden and mortality in rural Kenya. However, to our knowledge, the cost-effectiveness of these interventions has not yet been shown. To our knowledge, no evidence of the cost-effectiveness of group medical visits, microfinance, or a combination of group medical visits and microfinance (GMV–MF) to improve hypertension care in low-income and middle-income countries (LMICs) exists.

### Added value of this study

We used a model-based approach to estimate the incremental cost-effectiveness of group medical visits and microfinance interventions compared with usual care for participants in the BIGPIC hypertension trial in rural Kenya. We found that group medical visits and GMV–MF were likely to be incrementally cost-effective strategies to improve blood-pressure control in rural communities in Kenya. Furthermore, we estimated the value of adapting interventions by gender. We found that, due to the differential effectiveness of interventions by gender, group medical visits were estimated to be cost-effective for women but not for men, whereas GMV–MF was estimated to be cost-effective for women and men. To our knowledge, our analysis is one of the first to establish the value of group medical visits, microfinance, and GMV–MF in LMICs.

### Implications of all the available evidence

Our findings support the integration of BIGPIC features (ie, group medical visits and GMV–MF) into standard care for hypertension in Kenya and suggest that interventions should be adapted by gender. Our findings could improve the delivery, equity, and accessibility of hypertension-management resources, especially for rural populations, and reduce preventable cardiovascular incidents and deaths. Moreover, as BIGPIC used existing health-care infrastructure, our findings could be generalisable to other LMICs with similar health settings or populations.
