# Supplementary material for: Cost-effectiveness of group medical visits and microfinance interventions versus usual care to manage hypertension in Kenya: a secondary modelling analysis of data from the Bridging Income Generation with Group Integrated Care (BIGPIC) trial
Source: Lancet Glob Health. Author manuscript; Available in PMC 2024 Aug 7. (PMC11303878; doi:10.1016/S2214-109X(24)00188-8)
Supplement: 1 [file NIHMS2010732-supplement-1.pdf]

THE LANCET
Global Health

Supplementary appendix

This appendix formed part of the original submission and has been peer reviewed. We post it as supplied by the authors.

Supplement to: Chay J, Su RJ, Kamano JH, et al. Cost-effectiveness of group medical visits and microfinance interventions versus usual care to manage hypertension in Kenya: a secondary modelling analysis of data from the Bridging Income Generation with Group Integrated Care (BIGPIC) trial. *Lancet Glob Health* 2024; **12:** e1331–42.

Supplementary Material

Junxing Chay, Rebecca J. Su, Jemima H. Kamano, Benjamin Andama, Gerald S. Bloomfield, Allison K. Delong, Carol R. Horowitz, Diana Menya, Richard Mugo, Vitalis Orango, Sonak D. Pastakia, Cleophas Wanyonyi, Rajesh Vedanthan, Eric A. Finkelstein

Contents

Appendix S1 CHEERS 2022 Checklist[1] 3

Appendix S2 BIGPIC Study Data Vetting 6

Appendix S3 Computation of annual probabilities of first CVD events 7

Appendix S4 Annual probabilities of first to subsequent CVD events 8

Appendix S5 Costs of interventions, hypertension and chronic CVD management 11

Appendix S6 Disability weights 12

Appendix S7 Cost-effectiveness Planes 13

Appendix S8 Results of scenario analyses 14

Appendix S9 Tornado diagram of the ten most influential model parameters in the base case analysis using WTP threshold of 1X and 3X the GDP per capita 15

Appendix S1 CHEERS 2022 Checklist[1]

Topic	No.	Item	Location where item is reported
**Title**			
	1	Identify the study as an economic evaluation and specify the interventions being compared.	Title, Page 1
**Abstract**			
	2	Provide a structured summary that highlights context, key methods, results, and alternative analyses.	Abstract, Page 2
**Introduction**			
**Background and objectives**	3	Give the context for the study, the study question, and its practical relevance for decision making in policy or practice.	Introduction, Page 3
**Methods**			
**Health economic analysis plan**	4	Indicate whether a health economic analysis plan was developed and where available.	Not reported
**Study population**	5	Describe characteristics of the study population (such as age range, demographics, socioeconomic, or clinical characteristics).	Methods, Paragraph 8
**Setting and location**	6	Provide relevant contextual information that may influence findings.	Methods, Paragraphs 1 - 6
**Comparators**	7	Describe the interventions or strategies being compared and why chosen.	Methods, Paragraphs 1 - 6
**Perspective**	8	State the perspective(s) adopted by the study and why chosen.	Introduction, Paragraph 4
**Time horizon**	9	State the time horizon for the study and why appropriate.	Methods, Paragraph 7
**Discount rate**	10	Report the discount rate(s) and reason chosen.	Methods, Paragraph 9
**Selection of outcomes**	11	Describe what outcomes were used as the measure(s) of benefit(s) and harm(s).	Methods, Paragraph 9
**Measurement of outcomes**	12	Describe how outcomes used to capture benefit(s) and harm(s) were measured.	Methods, Paragraphs 16 - 17
**Valuation of outcomes**	13	Describe the population and methods used to measure and value outcomes.	Methods, Paragraphs 16 - 17

Topic	No.	Item	Location where item is reported
**Measurement and valuation of resources and costs**	14	Describe how costs were valued.	Methods, Paragraph 16
**Currency, price date, and conversion**	15	Report the dates of the estimated resource quantities and unit costs, plus the currency and year of conversion.	Methods, Paragraph 16
**Rationale and description of model**	16	If modelling is used, describe in detail and why used. Report if the model is publicly available and where it can be accessed.	Methods, Paragraphs 7 - 10
**Analytics and assumptions**	17	Describe any methods for analysing or statistically transforming data, any extrapolation methods, and approaches for validating any model used.	Methods, Paragraph 13 - 15
**Characterising heterogeneity**	18	Describe any methods used for estimating how the results of the study vary for subgroups.	Methods, Paragraph 18
**Characterising distributional effects**	19	Describe how impacts are distributed across different individuals or adjustments made to reflect priority populations.	Discussion
**Characterising uncertainty**	20	Describe methods to characterise any sources of uncertainty in the analysis.	Methods, Paragraph 19
**Approach to engagement with patients and others affected by the study**	21	Describe any approaches to engage patients or service recipients, the general public, communities, or stakeholders (such as clinicians or payers) in the design of the study.	Methods, Paragraph 1
**Results**			
**Study parameters**	22	Report all analytic inputs (such as values, ranges, references) including uncertainty or distributional assumptions.	Table 1
**Summary of main results**	23	Report the mean values for the main categories of costs and outcomes of interest and summarise them in the most appropriate overall measure.	Table 2
**Effect of uncertainty**	24	Describe how uncertainty about analytic judgments, inputs, or projections affect findings. Report the effect of choice of discount rate and time horizon, if applicable.	Results, Paragraphs 4 - 7
**Effect of engagement with patients and others affected by the study**	25	Report on any difference patient/service recipient, general public, community, or stakeholder involvement made to the approach or findings of the study	Discussion
**Discussion**			
**Study findings, limitations, generalisability, and current knowledge**	26	Report key findings, limitations, ethical or equity considerations not captured, and how these could affect patients, policy, or practice.	Discussion

Topic	No.	Item	Location where item is reported
**Other relevant information**			
**Source of funding**	27	Describe how the study was funded and any role of the funder in the identification, design, conduct, and reporting of the analysis	Declarations
**Conflicts of interest**	28	Report authors conflicts of interest according to journal or International Committee of Medical Journal Editors requirements.	Declarations

References

1. Husereau D, Drummond M, Augustovski F, de Bekker-Grob E, Briggs AH, Carswell C, et al. Consolidated Health Economic Evaluation Reporting Standards (CHEERS) 2022 Explanation and Elaboration: A Report of the ISPOR CHEERS II Good Practices Task Force. Value Health. 2022;25(1):10-31.

Appendix S2 BIGPIC Study Data Vetting

In order to maintain the integrity and reliability of the BIGPIC Study data and to ensure consistency across all the different study sites (counties), we implemented a robust combination of automated systems of data collection, validation and manual oversight, and continuous monitoring of key variables. This was informed by developing and following Standard Operating Procedures by the data management/analytic team.

During the data collection phases, both at baseline and follow-up assessments, the study used electronic data capture that assisted to minimize data entry and transcription errors. The field staff, (i.e. research assistants (RA)) received intensive training on data integrity, ethics of human subjects research, and practical sessions on using the data collection instruments. The Ras then used digital data collection devices (i.e. mobile phones/tablets) and the REDCap (Research Electronic Data Capture) mobile app. The REDCap app was programmed with built-in validation checks to minimize errors at the point of data entry. These tools were programmed to flag any inconsistent or missing data immediately.

Upon data synchronization onto the main BIGPIC research server, the data manager and team conducted periodic audits, which included cross-referencing a sample of entries with original source documents to verify accuracy and highlight discrepancies for action by the RAs. Rates of missingness and error rates were computed per RA or per region in order to establish which personnel or region needed further trainings to improve the data quality. Data summaries were performed periodically to identify the data trends, data quality issues like outliers, and logical inconsistencies in the datasets. Further, interim analyses were conducted on a regular basis and reported to the Data Safety and Monitoring Board, in order to monitor progress of the study and the outcomes. The results of the interim analysis were fed back to the data collection teams to address any abnormalities and to improve data collection processes.

Weekly data review meetings were held by the data management/analytic team and key team members as well as the RAs, to discuss any anomalies and to refine data collection and validation processes continuously. Feedback from these sessions was used to update training materials and SOPs, ensuring that data collectors remained informed of best practices and common errors.

The study also ensured all participants were provided with informed consent, and all institutional review board (IRB) requirements were adhered to. Data confidentiality was maintained by ensuring that strict protocols were followed in order to protect the data. The REDCap database was password-protected and could only be accessed by authorized personnel. Processed datasets for analyses were de-identified and validated for analysis.

By integrating mobile app technology, rigorous validation protocols, and a culture of continuous improvements, the BIGPIC Study maintained high standards of data quality, ensuring the reliability of data that informed these findings and reports.

Appendix S3 Computation of annual probabilities of first CVD events

We obtained post-trial QRISK®3 scores of MF, GMV and GMV-MF by subtracting respective difference-in-differences estimates (Table 1) from the QRISK®3 score of UC at baseline[1]. We used the following equation to derive annual probabilities of having a cardiac event after intervention from post-trial QRISK®3 scores. Following previous studies[2, 3], we assumed a constant hazard over ten years.

$$1 - \exp\left(0.1 \ln\left(1 - \frac{QRISK®3}{100}\right)\right)$$

Table S2 Annual probability of a first cardiac event after the BIGPIC intervention

Intervention	Post-trial QRISK®3 score	Annual probability of a first CVD event
**Population**		
UC	11.5	0.0121
MF	11.1	0.0117
GMV	11.2	0.0118
GMV-MF	10.6	0.0111
**Men**		
UC	11.9	0.0126
MF	11.8	0.0125
GMV	12.3	0.0130
GMV-MF	11.2	0.0118
**Women**		
UC	11.3	0.0119
MF	10.8	0.0113
GMV	10.7	0.0113
GMV-MF	10.3	0.0108

UC usual care; GMV group medical visits; MF microfinance

References

1. Vedanthan R, Kamano JH, Chrysanthopoulou SA, Mugo R, Andama B, Bloomfield GS, et al. Group Medical Visit and Microfinance Intervention for Patients With Diabetes or Hypertension in Kenya. J Am Coll Cardiol. 2021;77(16):2007-18.
2. Dixon P, Hollinghurst S, Ara R, Edwards L, Foster A, Salisbury C. Cost-effectiveness modelling of telehealth for patients with raised cardiovascular disease risk: evidence from a cohort simulation conducted alongside the Healthlines randomised controlled trial. BMJ Open. 2016;6(9):e012355.
3. Yang W, Gage H, Jackson D, Raats M. The effectiveness and cost-effectiveness of plant sterol or stanol-enriched functional foods as a primary prevention strategy for people with cardiovascular disease risk in England: a modeling study. Eur J Health Econ. 2018;19(7):909-22.

Appendix S4 Annual probabilities of first to subsequent CVD events

Table S3 shows the probabilities of having a fatal or non-fatal first and subsequent CVD event (heart attack and/or stroke) for the population as a whole and for each gender. Probabilities of first CVD events from Table S2 were disaggregated into the risk of a fatal or non-fatal specific event using assumed distribution and mortality data for each condition in Kenya. Probabilities of fatal and non-fatal first events are shown in the table below.

To obtain probabilities of a subsequent event, we used hazard ratios obtained from the literature to adjust probabilities of a first heart attack or stroke. These hazard ratios compared the risk of a CVD event for those with CVD history (heart attack/stroke/both) relative to those without. These HRs were adjusted for confounders including diastolic blood pressure, baseline systolic blood pressure, heart rate, age, sex, BMI, renal function, geographical region, physical activity, formal education, alcohol consumption, tobacco use, history of hypertension, myocardial infarction, stroke, transient ischemic attack, heart rhythm, comedications, study and study medications.[1] Using the formula in Appendix S2, we first converted probabilities to rates, multiplied the rates by relevant hazard ratios and converted adjusted rates back to probabilities, assuming a constant hazard over ten years.

Table S3 Annual probabilities of first to subsequent CVD events

Intervention/ events	Adjusted HRs	Population	Men	Women
**UC**				
First events				
Heart attack	-	0.0049	0.0050	0.0048
Stroke	-	0.0073	0.0076	0.0072
Fatal heart attack	-	0.0012	0.0013	0.0012
Fatal stroke	-	0.0033	0.0034	0.0032
Subsequent events				
Heart attack after one heart attack	1.42	0.0069	0.0071	0.0068
Heart attack after one stroke	1.00	0.0049	0.0050	0.0048
Stroke after one stroke	2.89	0.0209	0.0217	0.0205
Stroke after one heart attack	1.00	0.0073	0.0076	0.0072
Heart attack after one stroke and one heart attack	1.95	0.0094	0.0098	0.0093
Stroke after one stroke and one heart attack	3.13	0.0226	0.0235	0.0222
Fatal heart attack after one heart attack	1.22	0.0015	0.0015	0.0015
Fatal heart attack after one stroke	1.00	0.0012	0.0013	0.0012
Fatal stroke after one stroke	1.22	0.0040	0.0041	0.0039
Fatal stroke after one heart attack	1.00	0.0033	0.0034	0.0032
**MF**				
First events				
Heart attack	-	0.0047	0.0050	0.0045
Stroke	-	0.0070	0.0075	0.0068
Fatal heart attack	-	0.0012	0.0012	0.0011
Fatal stroke	-	0.0032	0.0034	0.0031
Subsequent events				

Heart attack after one heart attack	1.42	0.0066	0.0071	0.0064
Heart attack after one stroke	1.00	0.0047	0.0050	0.0045
Stroke after one stroke	2.89	0.0201	0.0214	0.0195
Stroke after one heart attack	1.00	0.0070	0.0075	0.0068
Heart attack after one stroke and one heart attack	1.95	0.0091	0.0097	0.0088
Stroke after one stroke and one heart attack	3.13	0.0218	0.0232	0.0212
Fatal heart attack after one heart attack	1.22	0.0014	0.0015	0.0014
Fatal heart attack after one stroke	1.00	0.0012	0.0012	0.0011
Fatal stroke after one stroke	1.22	0.0038	0.0041	0.0037
Fatal stroke after one heart attack	1.00	0.0032	0.0034	0.0031
**GMV**				
First event				
Heart attack	-	0.0047	0.0052	0.0045
Stroke	-	0.0071	0.0078	0.0068
Fatal heart attack	-	0.0012	0.0013	0.0011
Fatal stroke	-	0.0032	0.0035	0.0030
Subsequent event				
Heart attack after one heart attack	1.42	0.0067	0.0074	0.0064
Heart attack after one stroke	1.00	0.0047	0.0052	0.0045
Stroke after one stroke	2.89	0.0203	0.0224	0.0194
Stroke after one heart attack	1.00	0.0071	0.0078	0.0068
Heart attack after one stroke and one heart attack	1.95	0.0092	0.0101	0.0088
Stroke after one stroke and one heart attack	3.13	0.0219	0.0243	0.0210
Fatal heart attack after one heart attack	1.22	0.0014	0.0016	0.0014
Fatal heart attack after one stroke	1.00	0.0012	0.0013	0.0011
Fatal stroke after one stroke	1.22	0.0039	0.0043	0.0037
Fatal stroke after one heart attack	1.00	0.0032	0.0035	0.0030
**GMV-MF**				
First event				
Heart attack	-	0.0044	0.0047	0.0043
Stroke	-	0.0067	0.0071	0.0065
Fatal heart attack	-	0.0011	0.0012	0.0011
Fatal stroke	-	0.0030	0.0032	0.0029
Subsequent event				
Heart attack after one heart attack	1.42	0.0063	0.0067	0.0061
Heart attack after one stroke	1.00	0.0044	0.0047	0.0043
Stroke after one stroke	2.89	0.0191	0.0203	0.0186
Stroke after one heart attack	1.00	0.0067	0.0071	0.0065
Heart attack after one stroke and one heart attack	1.95	0.0086	0.0092	0.0084
Stroke after one stroke and one heart attack	3.13	0.0138	0.0147	0.0135
Fatal heart attack after one heart attack	1.22	0.0014	0.0014	0.0013

Fatal heart attack after one stroke	1.00	0.0011	0.0012	0.0011
Fatal stroke after one stroke	1.22	0.0037	0.0039	0.0036
Fatal stroke after one heart attack	1.00	0.0030	0.0032	0.0029

CVD cardiovascular disease; HR hazard ratio; UC usual care; GMV group medical visits; MF microfinance

References

1. Bohm M, Schumacher H, Teo KK, Lonn EM, Lauder L, Mancia G, et al. Cardiovascular outcomes in patients at high cardiovascular risk with previous myocardial infarction or stroke. J Hypertens. 2021;39(8):1602-10.

Appendix S5 Costs of interventions, hypertension and chronic CVD management

Table 4.1 Cost of intervention

	Cost per capita		Cost per health facility catchment area	
	First year	Subsequent years	First year	Subsequent years
**UC (n=118)**	**$87**	**$67**	**$10,238**	**$7,862**
Contracted	$85	$66	$10,095	$7,775
Labour	$1	$1	$143	$88
**MF (n=119)**	**$120**	**$67**	**$14,172**	**$7,946**
Contracted	$118	$66	$13,979	$7,830
Labour	$2	$1	$193	$116
**GMV (n=123)**	**$99**	**$71**	**$12,268**	**$8,749**
Contracted	$97	$69	$11,926	$8,473
Labour	$3	$2	$342	$276
**GMV-MF (n=122)**	**$139**	**$72**	**$16,913**	**$8,832**
Contracted	$136	$70	$16,532	$8,529
Labour	$3	$2	$380	$303

UC usual care; GMV group medical visits; MF microfinance. Costs are reported in 2020 US$.
First year costs are based on all contracted and labour costs incurred in the BIGPIC trial: administration and oversight, clinician and field staff training, participant training, baseline screening, confirmatory testing, intervention implementation, quarterly reviews and usual care activities. Subsequent year costs do not include costs of training, baseline screening, or confirmatory testing.

Table 4.2 Cost of hypertension and chronic CVD management

Parameter	Cost	Recurrent costs
**Hypertension management**	**$68**	**$68**
Medication	$60	$60
Clinic visits	$5	$5
Laboratory tests	$1	$1
**Chronic CVD management**	**$125**	**$125**
Medication	$100	$100
Clinic visits	$10	$10
Laboratory tests	$5	$5
Electrocardiogram	$10	$10

CVD cardiovascular disease. Costs are reported in 2020 US$

Appendix S6 Disability weights

Health state disability weights selected from GBD 2019 are presented below[1]. When more than one disability weight was appropriate for a health state, we generated a composite disability weight using estimation methods described below.

Table S5 Health states and their assigned disability weights from GBD 2019

Health state	Sequela	DW	Estimation Method
No CVD	Controlled, medically managed heart failure due to Pulmonary Arterial Hypertension (systemic hypertension, ..)	0.049	We weighed each DW by the prevalence of hypertension and diabetes at baseline as reported by the BIGPIC trial
	Uncomplicated diabetes mellitus type 1	0.049	
Chronic CVD due to one heart attack	Moderate angina due to ischemic heart disease	0.08	Simple average
	Moderate heart failure due to ischemic heart disease	0.07	
Chronic CVD due to one stroke	Chronic ischemic stroke severity level 1, mild	0.02	Simple average
	Chronic ischemic stroke severity level 2, moderate	0.07	
	Chronic ischemic stroke severity level 3, moderate plus cognition problems	0.32	
Chronic CVD due to two heart attacks	Severe angina due to ischemic heart disease	0.17	Simple average
	Severe heart failure due to ischemic heart disease	0.18	
Chronic CVD due to two strokes	Chronic ischemic stroke severity level 3, moderate plus cognition problems	0.32	Simple average
	Chronic ischemic stroke severity level 4, severe	0.55	
Chronic CVD due to one heart attack and stroke	-	0.33	Simple average of Chronic CVD due to 2 MIs and Chronic CVD due to 2 strokes.
Heart attack event	Acute myocardial infarction first 2 days	0.43	We weighed the first DW by $\frac{2}{365.4}$ and the second DW by $\frac{26}{365.4}$ to obtain a DW of 0.008 to account for the duration of the event.
	Acute myocardial infarction 3 to 28 days	0.07	
Stroke event	Acute ischemic stroke severity level 1, mild	0.02	We took the mean of these DWs to obtain 0.135. We then weighed this by $\frac{14}{365.4}$ to obtain a DW of 0.005 to account for the duration of the event.
	Acute ischemic stroke severity level 2, moderate	0.07	
	Acute ischemic stroke severity level 3, moderate plus cognition problems	0.32	

DW disability weight; CVD cardiovascular disease

References

1. Global Burden of Disease Collaborative Network. Global Burden of Disease Study 2019 (GBD 2019) Disability Weights. Seattle, United States of America: Institute for Health Metrics and Evaluation (IHME); 2020.

Appendix S7 Cost-effectiveness Planes

Figure S6.1 Cost-effectiveness plane for the population as a whole

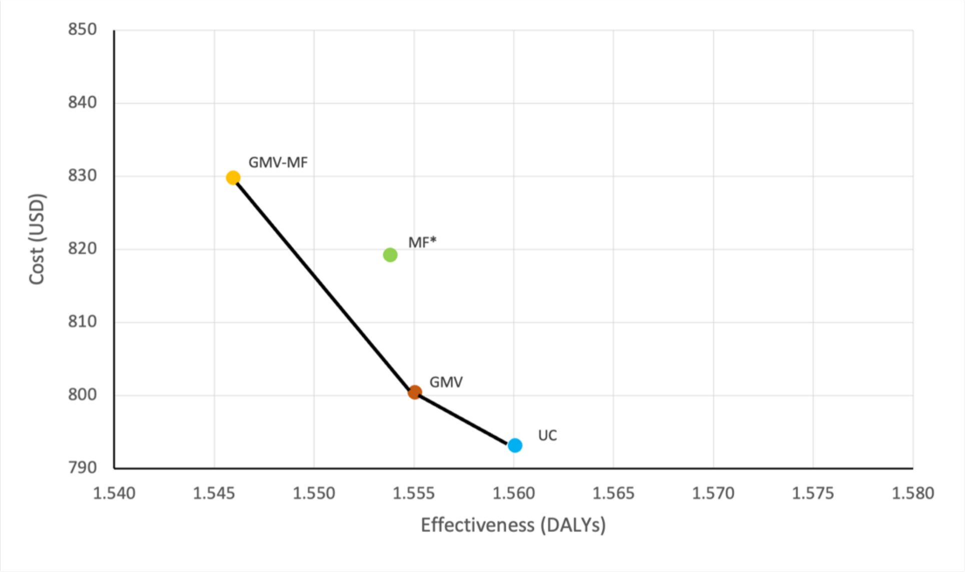

Figure S6.2 Cost-effectiveness plane for men and women

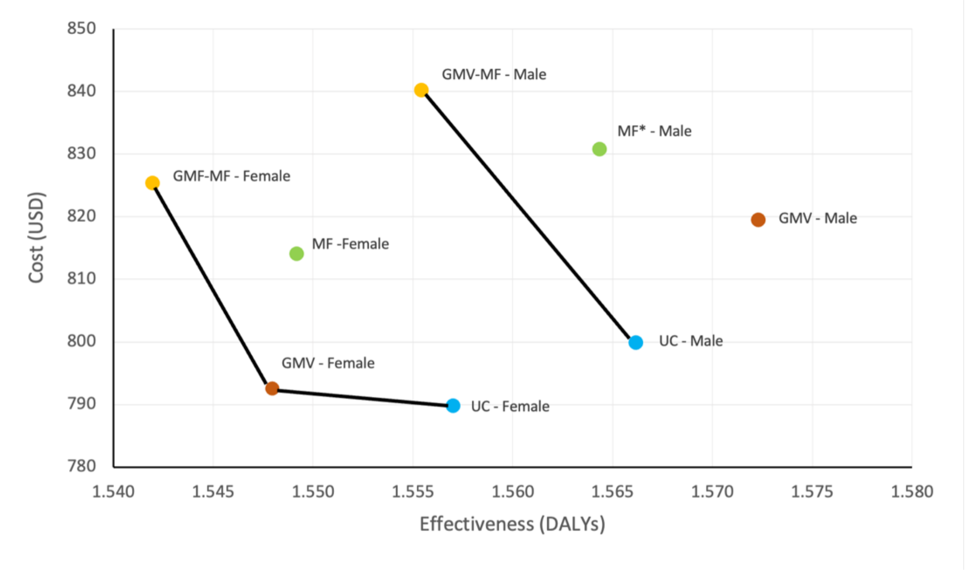

UC usual care; GMV group medical visits; MF microfinance; DALYs disability-adjusted life years
Black lines connecting interventions make up the cost-effectiveness frontier. All interventions on the cost-effectiveness frontier are non-dominated while all interventions not on the frontier are dominated or extended dominated. For an intervention to be extended dominated (interventions marked with asterisks) means that some combination of other interventions will be cheaper and more effective than that intervention.

Appendix S8 Results of scenario analyses

Intervention	Total cost	Incremental cost	Total DALYs	Incremental DALYs averted	ICERs
**20-year time horizon**					
UC	$1,134	-	4.755	-	-
GMV	$1,139	$5	4.743	0.013	$372
MF	$1,157	$18	4.740	0.003	Extended dominated
GMV-MF	$1,163	$25	4.720	0.023	$1,078
**Recurrent intervention costs**					
UC	$1,295	-	1.560	-	-
MF	$1,325	$30	1.554	0.006	$4,868
GMV	$1,335	$10	1.556	-0.001	Dominated
GMV-MF	$1,377	$52	1.546	0.008	$6,634

DALY disability-adjusted life year; ICER incremental cost-effectiveness ratio; UC usual care; GMV group medical visits; MF microfinance. Costs are reported in 2020 US$ on a per capita basis.

Appendix S9 Tornado diagram of the ten most influential model parameters in the base case analysis using WTP threshold of 1X and 3X the GDP per capita

Panel C: GMV vs. UC (1X GDP per capita)

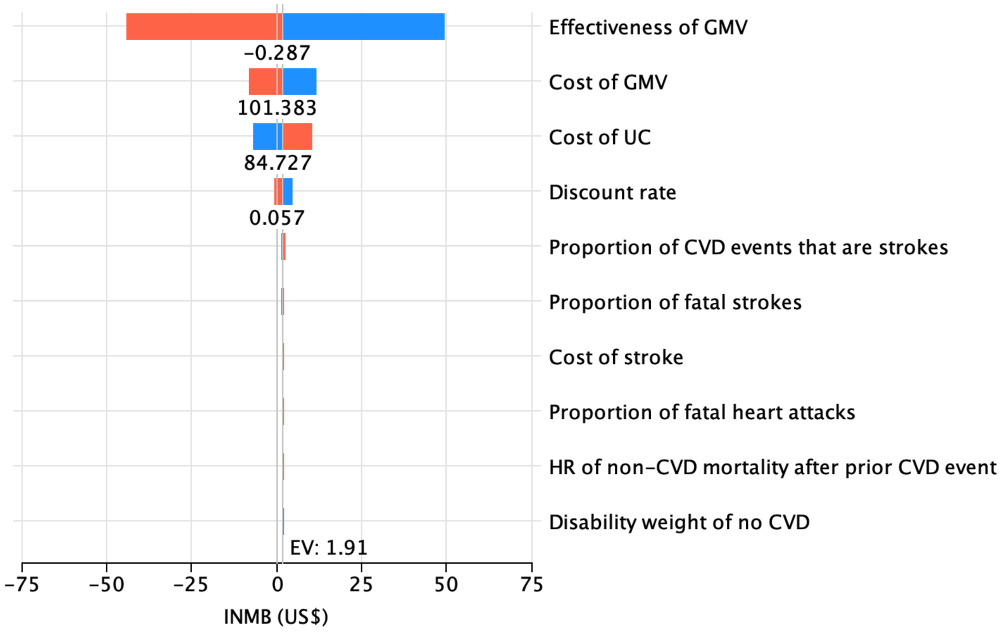

Panel D: GMV-MF vs. GMV (1X GDP per capita)

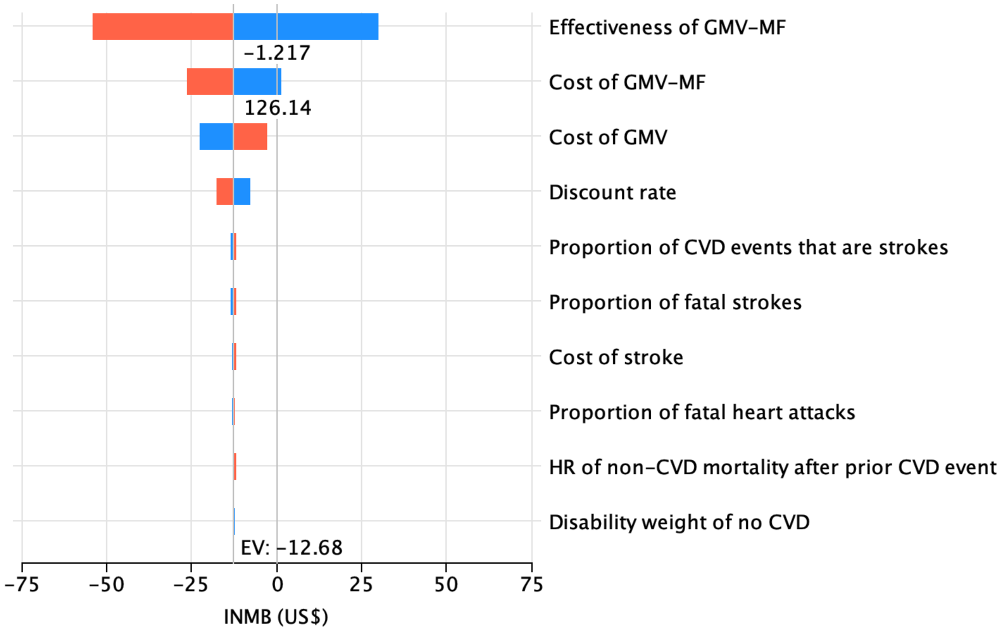

Panel E: GMV vs. UC (3X GDP per capita)

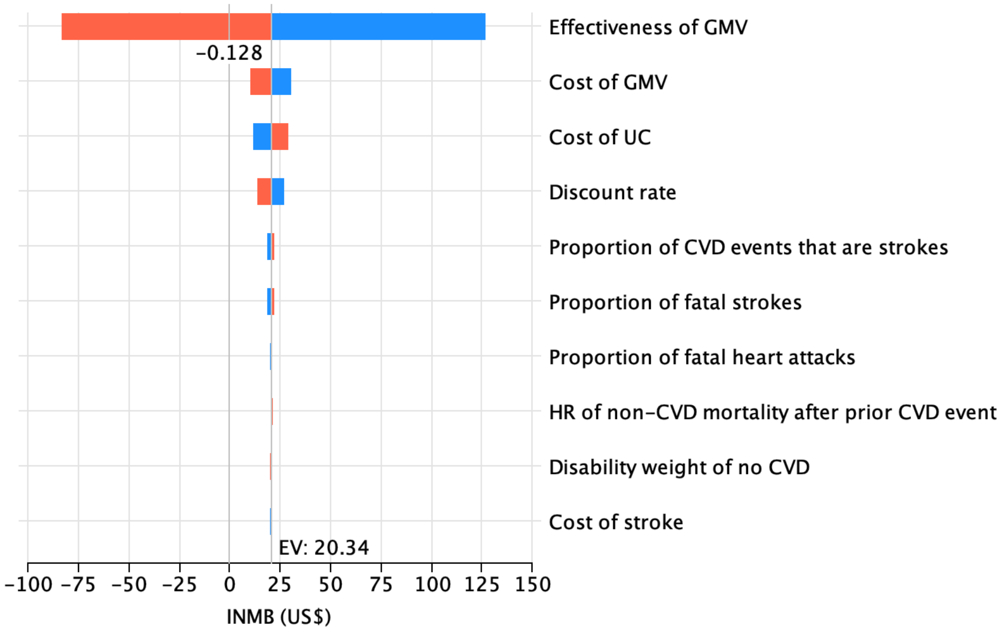

Panel F: GMV-MF vs. GMV (3X GDP per capita)

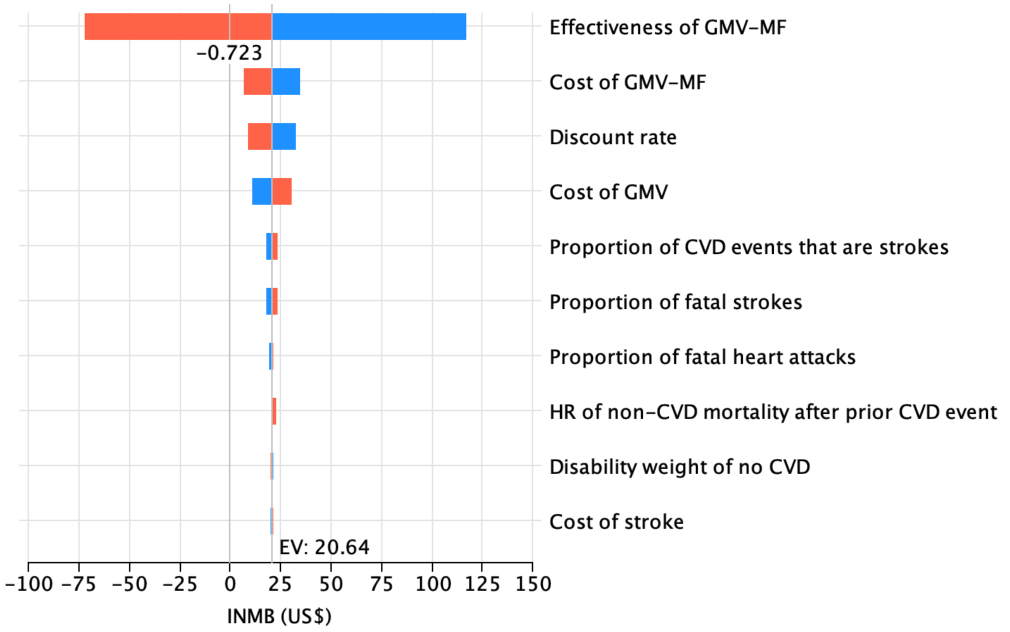

Sensitivity ranges are reported in Table 1. Using the threshold of 1X and 3X the GDP per capita of Kenya, Panels C, D and Panels E, F show the effects of parameter variation on INMBs (US$) for GMV relative to UC and GMV-MF relative to GMV. The bars indicate the range of INMB values corresponding to respective sensitivity ranges; the grey centre line indicates the base case INMB value; red and blue bars indicate when parameters are increasing and decreasing from their base case values, respectively.

INMB incremental net monetary benefit; UC usual care; GMV group medical visits; MF microfinance; CVD cardiovascular disease; HR hazard ratio; DALY disability-adjusted life year

References

1. Bohm M, Schumacher H, Teo KK, Lonn EM, Lauder L, Mancia G, et al. Cardiovascular outcomes in patients at high cardiovascular risk with previous myocardial infarction or stroke. J Hypertens. 2021;39(8):1602-10.
